# Halobacterium hubeiense sp. nov., a haloarchaeal species isolated from a bore core drilled in Hubei Province, PR China

**DOI:** 10.1099/ijsem.0.006296

**Published:** 2024-03-21

**Authors:** María José León, Cristina Sánchez-Porro, Rafael R. de la Haba, Friedhelm Pfeiffer, Mike Dyall-Smith, Hanna M. Oksanen, Antonio Ventosa

**Affiliations:** 1 Department of Microbiology and Parasitology, Faculty of Pharmacy, University of Sevilla, 41012 Sevilla, Spain; 2 Computational Biology Group, Max-Planck-Institute of Biochemistry, Martinsried, Germany; 3 Biology II, Ulm University, 89069 Ulm, Germany; 4 Veterinary Biosciences, Faculty of Veterinary and Agricultural Sciences, University of Melbourne, Parkville, VIC 3052, Australia; 5 Molecular and Integrative Biosciences Research Programme, Faculty of Biological and Environmental Sciences, University of Helsinki, Helsinki, Finland

**Keywords:** extremely halophilic archaea, halite, haloarchaea, *Halobacterium*, taxogenomics

## Abstract

Eight colonies of live microbes were isolated from an extensively surface-sterilized halite sample which had been retrieved from a depth of 2000 m from a salt mine in the Qianjiang Depression, Hubei Province, PR China. The eight colonies, obtained after 4 weeks of incubation, were named JI20-1^T^–JI20-8 and JI20-1^T^ was selected as the type strain. The strains have been previously described, including a genomic analysis based on the complete genome for strain JI20-1^T^ and draft genomes for the other strains. In that study, the name *Halobacterium hubeiense* was suggested, based on the location of the drilling site. Previous phylogenomic analysis showed that strain JI20-1^T^ is most closely related to the Permian isolate *Halobacterium noricense* from Alpine rock salt. The orthologous average nucleotide identity (orthoANI) and digital DNA–DNA hybridization (dDDH) percentages between the eight strains are 100–99.6 % and 99.8–96.4 %, respectively. The orthoANI and dDDH values of these strains with respect to the type strains of species of the genus *Halobacterium* are 89.9–78.2 % and 37.3–21.6 %, respectively, supporting their placement in a novel extremely halophilic archaeal species. The phylogenomic tree based on the comparison of sequences of 632 core-orthologous proteins confirmed the novel species status for these haloarchaea. The polar lipid profile includes phosphatidylglycerol, phosphatidylglycerol phosphate methyl ester, phosphatidylglycerol sulfate, and sulfated galactosyl mannosyl galactosyl glucosyl diether, a profile compatible with that of *Halobacterium noricense*. Based on genomic, phenotypic, and chemotaxonomic characterization, we propose strain JI20-1^T^ (=DSM 114402^T^ = HAMBI 3616^T^) as the type strain of a novel species in the genus *Halobacterium*, with the name *Halobacterium hubeiense* sp. nov.

## Introduction

Haloarchaea represent a large group of extremely halophilic archaea, constituting the class *Halobacteria* [1], within the phylum *Methanobacteriota* (formerly ‘*Euryarchaeota*’) [2]. Currently, the class *Halobacteria* includes two orders and nine families: the orders *Halobacteriales* (with families *Halobacteriaceae*, *Haladaptataceae*, *Haloarculaceae, Halococcaceae*, *Haloferacaceae*, *Natronoarchaeaceae*, *Natrialbaceae*, and *Halorubellaceae*) and *Halorutilales* (with the family *Halorutilaceae*), which include more than 70 genera [3,6]. The genus *Halobacterium*, together with *Halococcus*, were the first two haloarchaeal genera reported [7,8] and then later accepted as valid names in the Approved Lists of Bacterial Names [9]. At present, the genus *Halobacterium* is classified as the type genus of the family *Halobacteriaceae*, order *Halobacteriales*, within the class *Halobacteria* [10,11]. Currently, this genus includes eight species: *Halobacterium salinarum* (type species of the genus) [12,13], *Halobacterium bonnevillei* [14], *Halobacterium jilantaiense* [15], *Halobacterium litoreum* [16], *Halobacterium noricense* [13], *Halobacterium rubrum* [17], *Halobacterium wangiae* [18], and *Halobacterium zhouii* [18]. Species of *Halobacterium* are Gram-stain-negative, motile, rod-shaped cells, and their colonies are red or pink pigmented. They are aerobic, chemoorganotrophic, amino acids are required for growth, and sugars are poorly used. They are extremely halophilic, with growth occurring in media containing 15–32 % (w/v) NaCl, and optimal growth at 20–30 % (w/v) NaCl. The cells lyse in distilled water. The major polar lipids are phosphatidylglycerol (PG), phosphatidylglycerol phosphate methyl ester (PGP-Me), phosphatidylglycerol sulfate (PGS), and some glycolipids: galactosyl mannosyl glucosyl diether (TGD-1), sulfated galactosyl mannosyl glucosyl diether (S-TGD-1), or sulfated galactosyl mannosyl galactosyl glucosyl diether (S-TeGD) [10,11]. Their described DNA G+C content ranges from 54.3 to 71.2 mol% [10,11]. They have been isolated from water and sediment of salt lakes, solar salterns, saline soil, rock salt, salted fish and fish products, and salted hides [11,18].

Several studies have reported the isolation and characterization of strains of *Halobacterium* from halite crystals [13,19]. In 2016, Jaakkola *et al.* [20] isolated eight strains from a surface-sterilized 123 million-year-old bore core halite sample from a 2000 m deep salt mine in Qianjiang, Hubei Province, PR China. Data indicated that these new strains are closely related to *H. noricense*, a species isolated from an alpine Permian salt deposit in Austria [13]. The comparison of the complete genome sequence of the proposed type strain JI20-1^T^ and draft genomes of the other seven strains (JI20-2–JI20-8) with *H. noricense* indicated that they represent a novel species. The name ‘*Halobacterium hubeiense’* was suggested, but in the absence of a formal taxonomic description, the name has not been validated. In this study, we have carried out an exhaustive taxonomic characterization of the eight strains, including a genomic, phylogenetic, phenotypic, and chemotaxonomic characterization, and formally propose their classification as a new species, namely *Halobacterium hubeiense* sp. nov., based on the location of the drilling site.

## Methods

### New isolates and reference strains

The eight strains, designated as JI20-1^T^–JI20-8, were isolated previously from a rock salt drill core sample obtained at a depth of 2000 m from the northern part of the Qianjiang Depression (30 ° 29′ N 112 ° 58′ E) in Hubei Province, PR China [20]. These strains were isolated from surface-sterilized halite crystals, after dissolution in 20 % (w/v) artificial salt water and spread on Modified Growth Medium (MGM) plates, with 20 % (w/v) salts [21], and then incubated for up to 3 months at 37 °C under aerobic conditions. Details of the samples and isolation procedures used can be obtained from a previous publication [20].

In this study, the MGM medium with 25 % (w/v) salts was used for routine cultivation of these strains. The type strain of the species *H. salinarum* DSM 3754^T^, *H. noricense* DSM 15987^T^, and *Halorubrum saccharovorum* DSM 1137^T^ were used as reference strains in this study. They were grown on MGM medium with 25 % (w/v) salts, adjusted at pH 7.5, and incubated at 37 °C. They were long-term maintained in the same broth medium, with 20 % (v/v) glycerol at −80 °C.

### Phylogenetic 16S rRNA and phylogenomic analyses

The 16S rRNA gene sequence of strains JI20-1^T^−JI20-8 were compared with gene sequences from EzBioCloud database [22]. Using the arb software package [23] 16S rRNA gene alignments and phylogenetic trees were generated by three different algorithms: neighbour-joining [24], maximum-parsimony [25], and maximum-likelihood [26]. Bootstrap analysis (1000 pseudo-replicates) was performed to evaluate the robustness of the phylogenetic trees [27].

The Subcommittee on the Taxonomy of *Halobacteria* recommends the use of alternative phylogenetic markers such as the *rpoB*′ gene [28]. Therefore, *rpoB*′-based alignments and phylogenetic trees were reconstructed using BioEdit version 7.2.5 [29] and mega11 [30], respectively. Phylogenies were inferred using all the aforementioned algorithms and branch support was assessed by 1000 bootstrap resampling.

The genomic comparative analysis was carried out using the genome sequences of strains JI20-1^T^–JI20-8 obtained previously [20], and those of the type strains of species of *Halobacterium* and other genomes of related haloarchaeal species available in the GenBank database. The quality of the genome sequences studied was in agreement with the recommended minimal standards for the use of genome data for the taxonomy of prokaryotes [31]. Following these minimal standards, we used different overall genome relatedness indexes (OGRIs) to determine the status of strains JI20-1^T^–JI20-8 within the genus *Halobacterium*. Orthologous average nucleotide identity (OrthoANI) values were calculated using OAU software version 1.2 [32]. For digital DNA–DNA hybridization (dDDH), the Genome-to-Genome Distance Calculator bioinformatic tool (version 3.0) available from the Leibniz Institute DSMZ [33] was used. Finally, average amino acid identity (AAI) was calculated by the Enveomics tool [34].

The core genomes of the new isolates and other representative haloarchaea was determined by using, sequentially, the ‘rbm.rb’, ‘ogs.mcl.rb’, and ‘ogs.extract.rb’ scripts from the Enveomics suite [34]. In brief, clusters of orthologous genes (OGs), were identified by an all-*versus*-all blastp search based on the translated protein-coding gene sequences of our genome dataset. The OGs shared by all taxa and present in a single copy per genome were selected for further analysis. Translated single-copy core genes were aligned with muscle version 5.1 [35] and subsequently concatenated with the Enveomics script ‘Aln.cat.rb’ [34]. A maximum-likelihood phylogenomic tree was reconstructed using iq-tree version 2.2.7 [36] with the Q.pfam+F+I+R7 best-fit substitution model selected by ModelFinder [37] and branch support determined by ultrafast bootstrapping (1000 replicates) [38].

### Phenotypic characterization

The phenotypic characterization included morphological, physiological, biochemical, and nutritional features, and was performed following the minimal standards for the description of novel taxa of the class *Halobacteria* [39]. Cell morphology and motility of strains JI20-1^T^–JI20-8 were examined by a phase-contrast microscope (Zeiss Axioscope 5). The morphology of the colonies, their size, and pigmentation were observed on MGM medium with 25 % (w/v) salts after 20 days of incubation at 37 °C. Gram staining was determined by the Dussault method [40]. Optimal conditions for growth were determined by growing the strains in MGM medium with 0, 3, 5, 7.5, 10, 12.5, 15, 17.5, 20, 22.5, 25, and 30 % (w/v) NaCl. The growth of the isolates at different pH values was tested under the optimal salt concentration conditions, adjusting the pH of medium to pH 4.0, 5.0, 6.0, 7.0, 7.5, 8.0, 9.0, or 10.0 by adding appropriate buffers (MOPS, pH 4.0–7.0; Tris, pH 7.5–8.5; CHES, pH 9.0–10.0) to maintain stable pH values. The optimal growth temperature and its range were determined by incubating the strains at temperatures of 15, 20, 28, 30, 37, 40, 45, and 50 °C. All biochemical tests were carried out in MGM medium with 25 % (w/v) salts at 37 °C, unless otherwise stated. Growth under anaerobic conditions (with H_2_/CO_2_) was determined by incubation of strains in MGM medium with 25 % (w/v) salts in an anaerobic chamber using the Anaerogen system (Oxoid) to generate anaerobic atmosphere and an anaerobic indicator (Oxoid). Anaerobic growth using nitrate or dimethyl sulfoxide (DMSO) as alternative electron acceptors or by fermentation of arginine were determined as previously described [39]. Catalase activity was determined by adding a 3 % (v/v) H_2_O_2_ solution to colonies on MGM medium with 25 % (w/v) salts. Oxidase activity was examined with 1 % (v/v) tetramethyl-*p*-phenylenediamine [41]. Hydrolysis of aesculin, casein, gelatin, starch, and Tween 80, production of indole, and phosphatase activity were determined as described by Cowan and Steel [42]. The utilization of substrates as carbon and energy sources was determined by the classical method of Koser [43], as modified by Ventosa *et al.* [44]. Substrates were added as filter-sterilized solutions to give a final concentration of 1 g l^−1^, except for carbohydrates, which were used at 2 g l^−1^.

### Chemotaxonomic analysis

The polar lipids of strain JI20-1^T^, chosen as representative of the new isolates, and the reference strains *H. salinarum* DSM 3754^T^, *H. noricense* DSM 15987^T^, and *Halorubrum saccharovorum* DSM 1137^T^ were determined from cells cultured in MGM medium with 25 % (w/v) salts and incubated at 37 °C for 15 days. The total polar lipids were extracted as follows: the cell biomass was washed by adding 25 % (w/v) NaCl sterile solution and centrifuged for 1 min at 6000 *g*; the pellet was then resuspended in 0.8 ml of 25 % (w/v) NaCl solution until obtaining a cell suspension. To 0.8 ml cell suspension, 2 ml methanol and 1 ml chloroform were added to create a monophasic mixture. After gently mixing by inversion for 1 h and subsequent centrifugation for 1 min at 6000 *g*, the supernatant was collected from the colourless pellet. The supernatant in monophase was disrupted by adding 500 µl 0.2 M KCl and 1 ml chloroform, followed by centrifugation for 1 min at 6000 *g*. The lower pigmented phase of the bilayer phase, corresponding to the chloroform fraction, was recovered and concentrated to ~500 µl by evaporation under a fume hood or vacuum concentrator. The extract was transferred to a weighted empty glass vial of 2 ml, dried, weighted, and stored at −20 °C. To perform the chromatography, the total lipid extract was dissolved, resulting in a final concentration of 100 mg ml^−1^. A volume of 10 µl total lipid extract (100 mg ml^−1^) was analysed by high-performance thin-layer chromatography (HPTLC), using an HPTLC silica gel 60 plates crystal back (10,620 cm; Merck art. 5626). The plates were developed in the solvent system consisting of chloroform–methanol–90 % acetic acid (65 : 4 : 35) as previously described [45,46]. To detect all polar lipids, the plate was sprayed with 5 % (v/v) sulfuric acid in water and charred by heating at 160 °C. The phospholipids were revealed by molybdenum blue spray reagent [45].

## Results and discussion

### Single marker-based phylogenies and genomic analyses

**Fig. 1.**
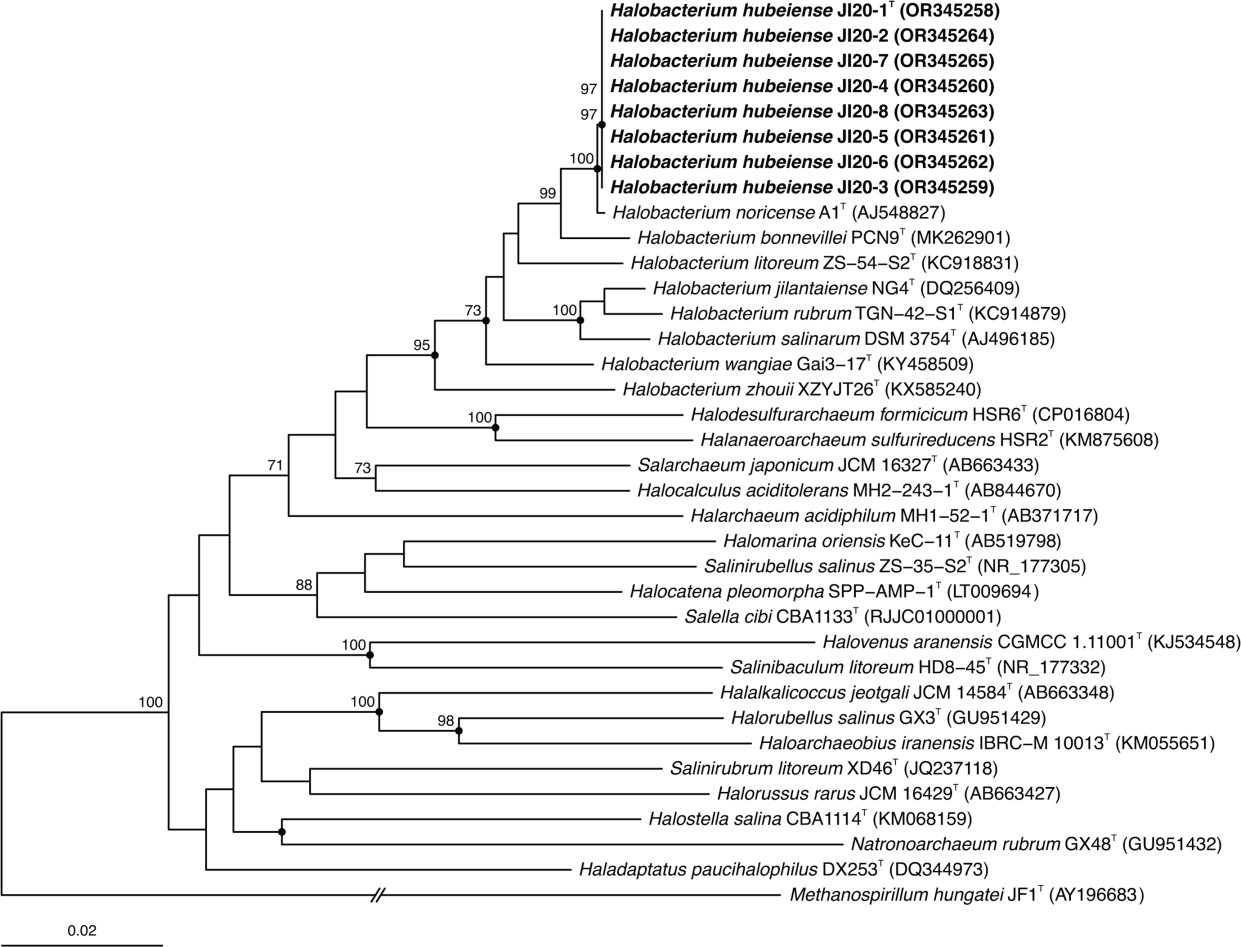
Neighbour-joining phylogenetic tree based on the comparison of the 16S rRNA gene sequences showing the relationship between strains JI20-1^T^–JI20-8, the type strains of species of the genus *Halobacterium,* and other related haloarchaeal genera. The sequence accession numbers are shown in parentheses. Bootstrap values equal or higher than 70 % are indicated above the nodes. Black circles indicate that the corresponding nodes were also obtained in the trees generated with the maximum-parsimony and maximum-likelihood algorithms. *Methanospirillum hungatei* JF1^T^ was used as outgroup. Bar, 0.02 substitutions per nucleotide position.

The 16S rRNA gene sequence identity among the eight strains investigated varied from 99.9 to 100 %. The 16S rRNA gene sequence identity between strain JI20-1^T^, chosen as representative of the eight new isolates, with respect to the type strain of the most closely related haloarchaea, species of the genus *Halobacterium*, was 99.9 % with *H. noricense* A1^T^, 98.6 % with *H. bonnevillei* PCN9^T^, 97.3 % with *H. jilantaiense* CGMCC 1.5337^T^, 97.3 % with *H. wangiae* Gai3-17^T^, 97.3 % with *H. litoreum* ZS-54-S2^T^, 97.1 % with *H. salinarum* DSM 3754^T^, 96.7 % with *H. rubrum* TNG-42-S1^T^, and 96.1 % with *H. zhouii* XZYJT26^T^, respectively. The percentages of identity with species of other haloarchaeal genera were equal or lower than 94.1 %. Phylogenetic tree reconstructions based on the 16S rRNA and *rpoB*′ gene sequences were conducted using the neighbour-joining [24], maximum-parsimony [25], and maximum-likelihood [26] algorithms and they confirmed the placement of the eight new isolates into a separate branch of species of *Halobacterium*, very closely related to the type strain of *H. noricense* A1^T^ (Figs1  2).

**Fig. 2.**
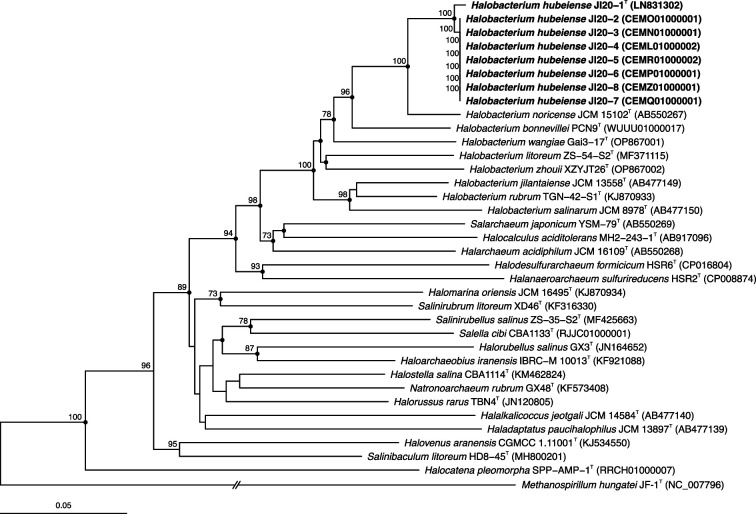
Neighbour-joining phylogenetic tree based on the comparison of the *rpoB*′ gene sequences showing the relationships between strains JI20-1^T^–JI20-8, the type strains of species of the genus *Halobacterium*, and other related haloarchaeal genera. The sequence accession numbers are shown in parentheses. Bootstrap values equal or higher than 70 % are indicated above the nodes. Black circles indicate that the corresponding nodes were also obtained in the trees generated with the maximum-parsimony and maximum-likelihood algorithms. *Methanospirillum hungatei* JF1^T^ was used as outgroup. Bar, 0.05 substitutions per nucleotide position.

**Fig. 3.**
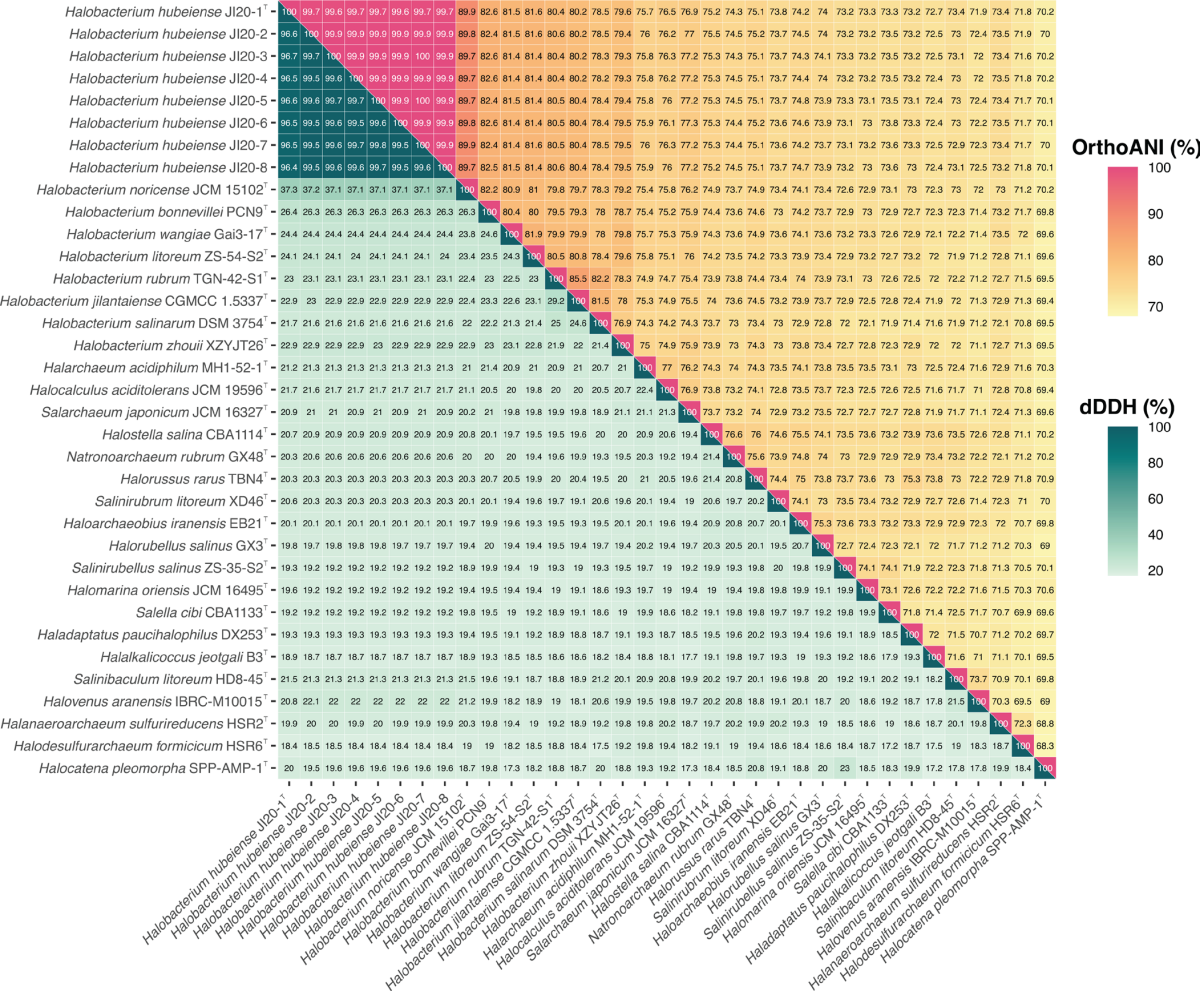
Orthologous average nucleotide Identity (OrthoANI) and digital DNA–DNA hybridization (dDDH) values (%) of strains JI20-1^T^–JI20-8, the type strains of species of the genus *Halobacterium*, and other related haloarchaeal genera.

**Fig. 4.**
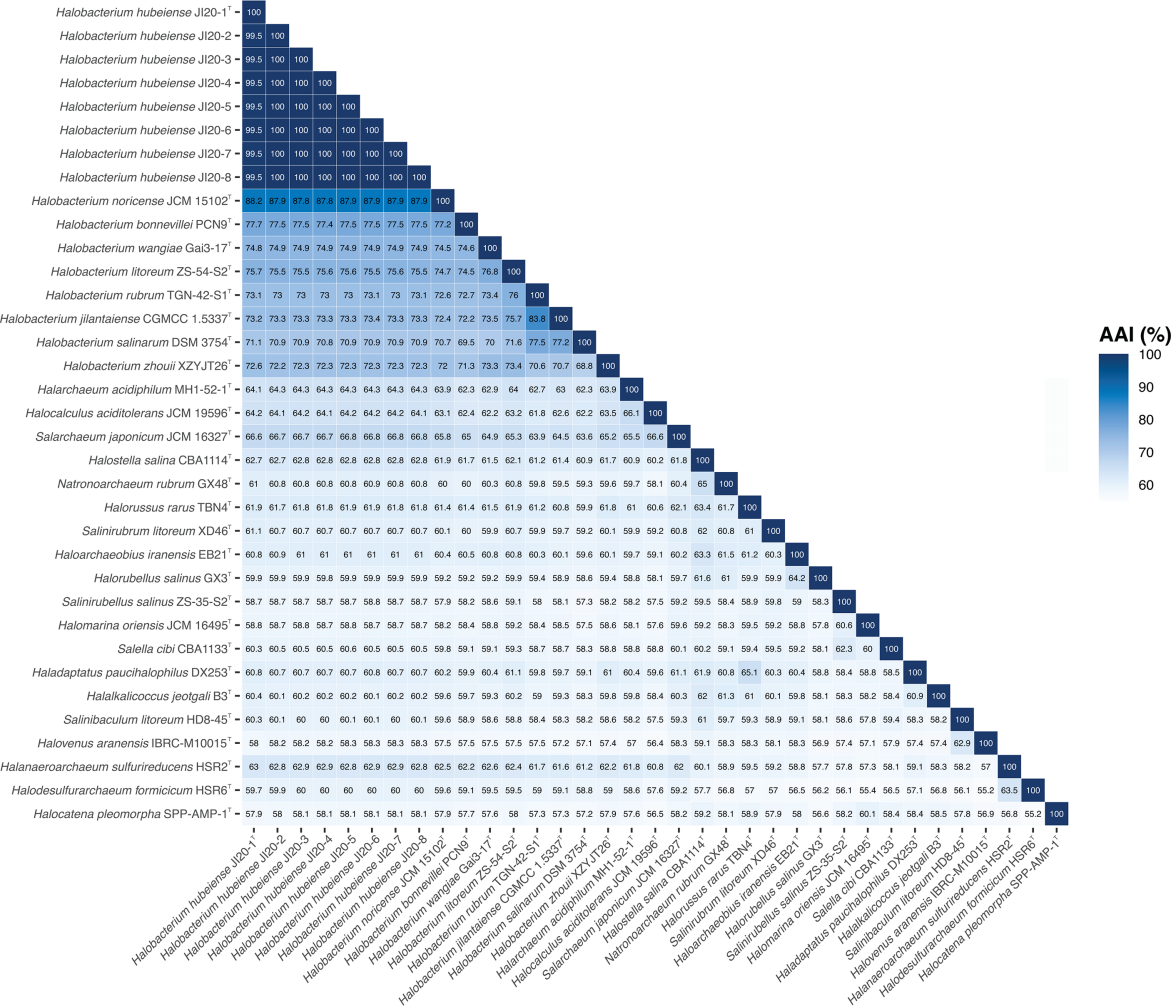
Average amino acid identity (AAI) percentages among strains JI20-1^T^ to JI20-8, the type strains of species of the genus *Halobacterium*, and other related haloarchaeal genera.

The previously reported genome sequences of the eight strains [20] enabled complete genomic and phylogenomic analyses of the strains with respect to other related type strains of haloarchaea with genomes available in public databases. We calculated the OGRIs currently used for taxonomic studies, namely orthologous average nucleotide identity (OrthoANI), digital DNA–DNA hybridization (dDDH), and average amino acid identity (AAI). The OrthoANI percentages between strain JI20-1^T^ and the other seven strains were 99.7–99.6 %, and those between the eight strains and the type strains of species of *Halobacterium* were 89.9 % (with *H. noricense* JCM 15102^T^) to 78.2 % (Fig. 3). The dDDH percentages among the eight strains were from 99.8 to 96.4 %, and those between these strains and the type strains of species of *Halobacterium* ranged from 21.6 to 37.3 % (Fig. 3). Finally, the AAI values determined among the eight strains were 99.5–100 %, and those between these strains and the type strains of species of *Halobacterium* were 70.8–88.2 % (Fig. 4). Therefore, the percentages obtained for the genomic indexes were within the accepted cutoff limits for prokaryotic species delineation: 95 % for ANI [47,50], 70 % for dDDH [50,51], and 95 % for AAI [52,53]. These results supported unambiguously the placement of the eight strains into a novel species of the genus *Halobacterium*.

**Fig. 5.**
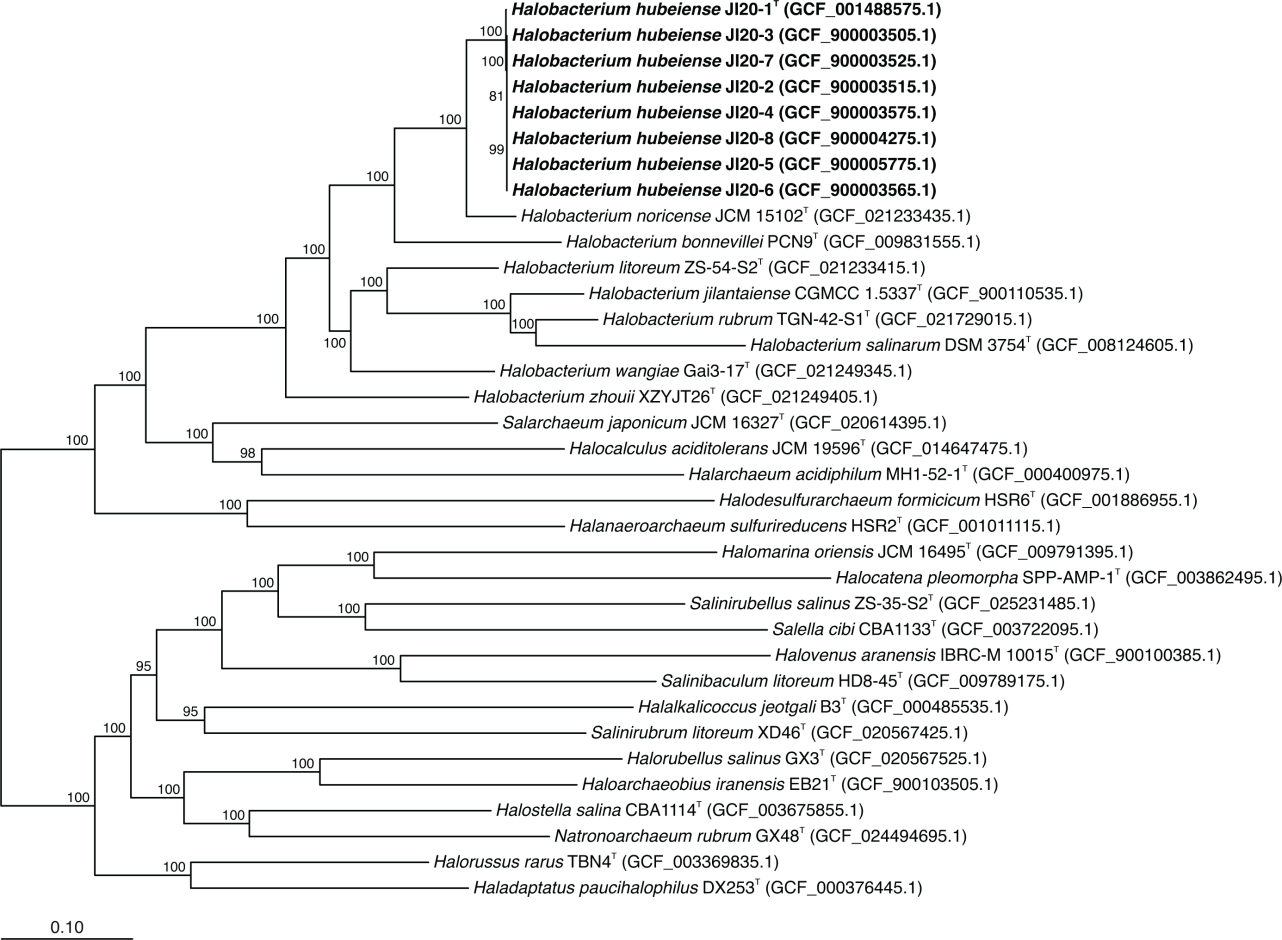
Maximum-likelihood phylogenomic tree reconstruction based on the 632 translated single-copy core orthologous genes of strains JI20-1^T^–JI20-8, the species of the genus *Halobacterium,* and representative species of genera of the family *Halobacteriaceae*. The genome sequence accession numbers are shown in parentheses. Bootstrap values equal or higher than 70 % are indicated at branch points. Bar, 0.10 substitutions per amino acid position.

A maximum-likelihood phylogenomic tree (Fig. 5) was reconstructed as indicated in the methods section, based on the sequence of 632 core-orthologous proteins. This tree shows that strains JI20-1^T^–JI20-8 cluster together, and separately from the species *H. noricense* and the other species of *Halobacterium*. In addition, the tree topology confirms the monophyletic structure of the genus *Halobacterium* within the family *Halobacteriaceae*, with *H. zhouii* as the most peripheral species of this genus (Fig. 5).

The sizes of the complete genome of strain JI20-1^T^ and draft genomes of strains JI20-2–JI20-8 are 3.1–3.2 Mb, and their genomic G+C contents are from 66.2 to 66.6 mol% [20]. This DNA G+C content is within the range of species of the genus *Halobacterium* [10,11]. The genome of strain JI20-1^T^ consists of one chromosome (2.5 Mb) with high G+C (68.4 mol%) and three plasmids (0.35, 0.16, and 0.11 Mb, respectively) with a lower G+C content (60.0, 59.3, and 56.5 mol%, respectively). A single rRNA operon and 47 tRNA genes were detected in the genome of strain JI20-1^T^. Additional information about the genome organization of *H. hubeiense* and comparative genomic analysis with *H. noricense* is available in Jaakkola *et al.* [20].

### Phenotypic characterization

The phenotypic features of the eight strains (JI20-1^T^–JI20-8) were quite similar. The cells appeared as rod shaped (0.4 µm wide and 1.0–1.5 µm long) occurring singly or in pairs, and motile. Cells formed regular, pale red pigmented colonies. Cells grew optimally in the presence of 22.5–25 % (w/v) NaCl and in the range 15–30 % (w/v) NaCl. On liquid medium, the strains could grow in the temperature range from 28 to 45 °C. Optimal growth was observed at 37–45 °C, but there was no growth at 50 °C. The strains grew optimally at pH 7.5–8.0 and were able to grow at pH 5.0–9.0. The strains are chemoorganotrophic and strictly aerobic and cannot grow anaerobically using nitrate, DMSO, or arginine. All eight strains were catalase positive and oxidase negative. They were able to hydrolyse aesculin and Tween 80, but not casein, gelatin, or starch. Indole and phosphatase were not produced. Other characteristics of the eight strains and the differential features between these strains and the type strains of the most closely related species *H. noricense* DSM 15987^T^ and the type species of the genus, *H. salinarum* DSM 3754^T^, are given in the species description and Table 1.

**Table 1.** Differential characteristics between the eight strains (JI20-1^T^ to JI20-8), the most closely related species *Halobacterium noricense,* and the type species of the genus *Halobacterium* Strains: 1, JI20-1^T^; 2, JI20-2; 3, JI20-3; 4, JI20-4; 5, JI20-5; 6, JI20-6; 7, JI20-7; 8, JI20-8; 9, *Halobacterium noricense* DSM 15987^T^; 10, *Halobacterium salinarum* DSM 3754^T^. All data are from this study unless otherwise indicated. +, Positive; −, negative.

Characteristic	1	2	3	4	5	6	7	8	9	10
Cell morphology	Single or paired rods	Single or paired rods	Single or paired rods	Single or paired rods	Single or paired rods	Single or paired rods	Single or paired rods	Single or paired rods	Single rods	Rods
Colony pigmentation	Pale red	Pale red	Pale red	Pale red	Pale red	Pale red	Pale red	Pale red	Red	Red-pink
Cell size (µm)	0.4×1.0–1.5	0.4×1.0–1.5	0.4×1.0–1.5	0.4×1.0–1.5	0.4×1.0–1.5	0.4×1.0–1.5	0.4×1.0–1.5	0.4×1.0–1.5	0.6×1.2–2.0*	0.5–1.0×1.0–6.0
NaCl range for growth (%, w/v)	15.0–30.0	15.0–30.0	15.0–30.0	15.0–30.0	15.0–30.0	15.0–30.0	15.0–27.5	15.0–27.5	12.5–25.0	17.5–30.0
NaCl optimum for growth (%, w/v)	22.5–25.0	22.5–25.0	22.5	22.5	22.5	22.5	22.5	22.5	15.0–17.5	20.5–25.0
Temperature range for growth (°C)	28–45	28–45	28–45	28–45	28–45	28–45	28–45	28–45	28–50	20–55
Temperature optimum for growth (°C)	37	37–45	37	37–45	37–45	37–45	37	37–45	45	37
pH range for growth	5.0–9.0	5.0–9.0	5.0–9.0	5.0–9.0	5.0–9.0	5.0–9.0	5.0–9.0	5.0–9.0	5.0–8.5	5.5–8.0
pH optimum for growth	7.5	7.5	7.5–8.0	7.5–8.0	8.0	8.0	7.5–8.0	7.5–8.0	7.0–7.5	7.0–7.5
Tween 80 hydrolysis	+	+	+	+	+	+	+	+	−	−
Anaerobic growth in the presence of:										
Nitrate	−	−	−	−	−	−	−	−	+	−
Arginine	−	−	−	−	−	−	−	−	+	+
Utilization of d-glucose	+	+	+	+	+	+	+	+	−	−
DNA G+C content (mol%) (genome)	66.6	66.2	66.3	66.3	66.4	66.3	66.3	66.2	64.8	66.3

*Data from Guber *et al*. [13].

†Data from Oren and Ventosa [11].

### Chemotaxonomic characterization

The polar lipids of strain JI20-1^T^, selected as representative of the eight isolates, were extracted and compared by HPTLC with those from the reference strains *H. salinarum* DSM 3754^T^, *H. noricense* DSM 15987^T^, and *Halorubrum saccharovorum* DSM 1137^T^. The major polar lipids of strain JI20-1^T^ were PG, PGP-Me, PGS, and one glycolipid chromatographically identical to S-TeGD (Fig. S1, available in the online version of this article). This polar lipid profile is similar to that of the most closely related species, *H. noricense* DSM 15987^T^ [13], in contrast to that of *H. salinarum* DSM 3754^T^, which included S-TGD-1 (Fig. S1) [10,11].

Overall, all data reported previously as well as in this study, based on the phylogenetic, genomic, phenotypic, and chemotaxonomic comparisons of the eight isolates with the species of the genus *Halobacterium*, support the placement of these isolates as a new species of the genus *Halobacterium*, for which we propose the name *Halobacterium hubeiense* sp. nov.

## Description of *Halobacterium hubeiense* sp. nov.

*Halobacterium hubeiense* (hu.bei.en’se. N.L. neut. adj. *hubeiense*, pertaining to the province of Hubei, where the bore core has been drilled from which the strain was isolated).

Cells are motile rod-shaped (0.4×1.0–1.5 µm) and Gram-stain-negative, occurring single or in pairs. Colonies on agar plates containing 25 % (w/v) NaCl are pale-red pigmented and round. Growth is chemoorganotrophic and aerobic. Growth occurs with 15–30 % (w/v) NaCl and 0.05–0.3 M Mg^2+^ at pH 5.0–9.0 and at 28–45 °C on liquid media (no growth at 50 °C). Optimal NaCl concentration, Mg^2+^ concentration, pH, and temperature for growth are 22.5–25.0 % (w/v), 0.1 M, pH 7.5–8.0, and 37–45 °C, respectively. Not able to grow under anaerobic conditions by fermentation of arginine. Not able to grow anaerobically using nitrate or DMSO. Bacteriorhodopsin is not produced. Catalase positive and oxidase negative. Able to hydrolyse Tween 80 and aesculin, but not casein, gelatin, or starch. Indole is not produced. Phosphatase test is negative. Able to utilize the following compounds as sole carbon and energy source: arabinose, cellobiose, d-glucose, lactose, mannose, propionate, and l-lysine, but not amygdalin, maltose, arbutin, xylose, dulcitol, mannitol, valerate, and citrulline. The major polar lipids are PG, PGP-Me, PGS, and S-TeGD. The whole genomic DNA G+C content is 66.6–66.2 mol%.

The type strain is JI20-1^T^ (=DSM 114402^T^ = HAMBI 3616^T^), which was isolated from a deep drilling bore core at Wangchang in Qianjiang Depression, Hubei Province, PR China. It was isolated from this bore core and, specifically, its subsection from 2026.51 to 2026.80 m consisting of crystal halite. The DNA G+C content of the type strain is 68.4 mol% (chromosome, 2.5 Mb) and in the range of 60.0–56.5 mol% for the three plasmids (0.62 Mb in total). Additional reference strains of this species are strains JI20-2–JI20-8.

The GenBank/EMBL/DDBJ accession number for the 16S rRNA gene sequence of strain JI20-1^T^ is OR345258 and that of the complete genome is GCF_001488575.1 (LN831302–LN831305).

## supplementary material

10.1099/ijsem.0.006296 Uncited Fig. S1.

## Abbreviations

AAI: average amino acid identity
dDDH: digital DNA–DNA hybridization
DMSO: dimethyl sulfoxide
OC: orthologous cluster
OrthoANI: orthologous average nucleotide identity
PG: phosphatidylglycerol
PGP-Me: phosphatidylglycerol phosphate methyl ester
PGS: phosphatidylglycerol sulfate
S-TeGD: sulfated galactosyl mannosyl galactosyl glucosyl diether
S-TGD-1: sulfated galactosyl mannosyl glucosyl diether
TGD-1: galactosyl mannosyl glucosyl diether
