# Supplementary material for: Halobacterium hubeiense sp. nov., a haloarchaeal species isolated from a bore core drilled in Hubei Province, PR China
Source: Int J Syst Evol Microbiol. 2024 Mar 21;74(3):006296. doi: 10.1099/ijsem.0.006296 (PMC11004503; doi:10.1099/ijsem.0.006296)
Supplement: Uncited Fig. S1. [file ijsem-74-06296-s001.pdf]

Supplementary Material

Halobacterium hubeiense sp. nov., a haloarchaeal species isolated from a bore core drilled in the Hubei Province, China

María José León[1], Cristina Sánchez-Porro[1], Rafael R. de la Haba[1], Friedhelm Pfeiffer[2,3], Mike Dyall-Smith[2,4], Hanna M. Oksanen[5]* and Antonio Ventosa[1]*

[1]Department of Microbiology and Parasitology, Faculty of Pharmacy, University of Sevilla, Sevilla, Spain.

[2]Computational Biology Group, Max-Planck-Institute of Biochemistry, Martinsried, Germany.

[3]Biology II, Ulm University, 89069 Ulm, Germany.

[4]Veterinary Biosciences, Faculty of Veterinary and Agricultural Sciences, University of Melbourne, Parkville, VIC 3052, Australia.

[5]Molecular and Integrative Biosciences Research Programme, Faculty of Biological and Environmental Sciences, University of Helsinki, Helsinki, Finland.

*Correspondence: hanna.oksanen@helsinki.fi, ventosa@us.es

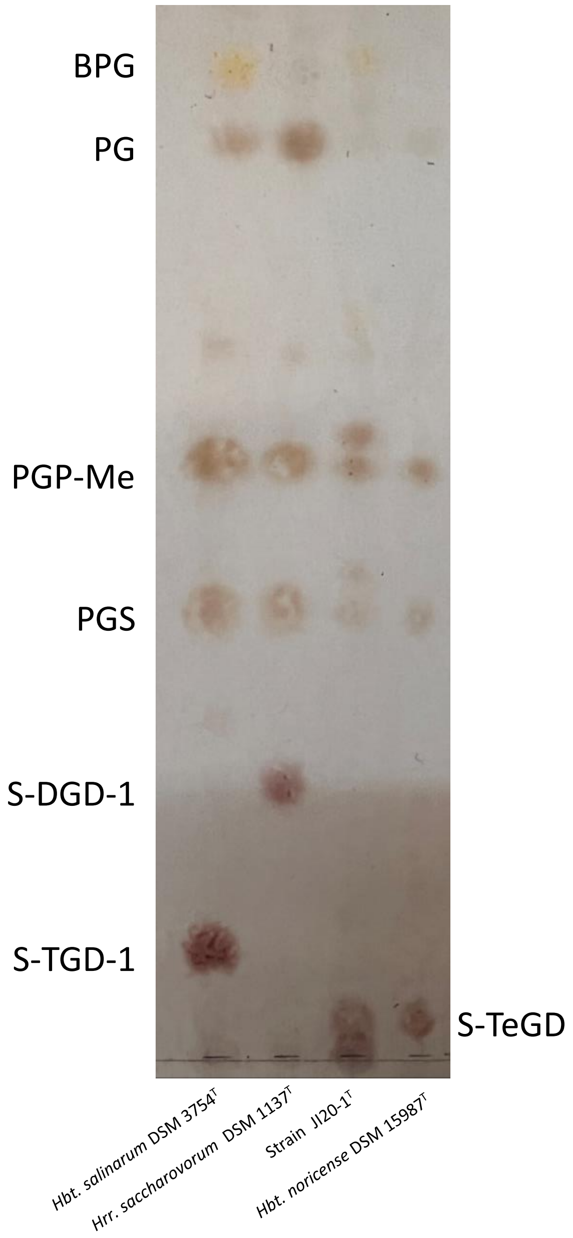

Supplementary Figure S1. High-Performance Thin Layer Chromatography (HPTLC) of polar lipids of strain JI20-1^T and some other reference haloarchaeal species. The plate was revealed with sulfuric acid 5 % (v/v) in water, followed by heating at 160 ºC. Lines: 1, *Halobacterium salinarum* DSM 3754^T; 2, *Halorubrum saccharovorum* DSM 1137^T; 3, Strain JI20-1^T: 4, *Halobacterium noricense* DSM 15987^T

Abbreviations: BPG, biphosphatidylglycerol; PG, phosphatidylglycerol; PGP-Me, phosphatidylglycerol phosphate methyl ester; PGS, phosphatidylglycerol sulfate; S-DGD-1, sulfated mannosyl glucosyl diether; S-TGD-1, sulfated galactosyl mannosyl glucosyl diether; S-TeGD, sulfated galactosyl mannosyl galactosyl glucosyl diether.
